# Molecular epidemiology and azole resistance mechanism study of *Candida guilliermondii* from a Chinese surveillance system

**DOI:** 10.1038/s41598-017-01106-7

**Published:** 2017-04-19

**Authors:** Jing-Wei Cheng, Kang Liao, Timothy Kudinha, Shu-Ying Yu, Meng Xiao, He Wang, Fanrong Kong, Ying-Chun Xu

**Affiliations:** 1 Department of Clinical Laboratory, Peking Union Medical College Hospital, Chinese Academy of Medical Sciences, Beijing, 100730 China; 2 Graduate School, Peking Union Medical College, Chinese Academy of Medical Sciences, Beijing, 100730 China; 3 Beijing Key Laboratory for Mechanisms Research and Precision Diagnosis of Invasive Fungal Diseases, Beijing, 100730 China; 4 Department of Laboratory Medicine, The First Affiliated Hospital of Sun Yat-sen University, Guangzhou, Guangdong 510080 China; 5 The Charles Sturt University, Leeds Parade, Orange, New South Wales, 2687 Australia; 6 Centre for Infectious Diseases and Microbiology Laboratory Services, Westmead Hospital, Westmead, New South Wales, 2145 Australia

## Abstract

We studied the molecular epidemiology and mechanism of azole resistance of 164 *C. guilliermondii* isolates from a nationwide multi-center surveillance program. The isolates were identified by ITS gene sequencing, and the *in vitro* susceptibility to fluconazole and voriconazole was determined by broth microdilution method. The 14-α-demethylase gene *ERG11* was amplified and sequenced, and microsatellite analysis was performed to study the genetic relatedness of the isolates. Amongst the 164 *C. guilliermondii* isolates, 15 (9.1%) and 17 (10.4%) isolates were assigned to be non-wild type (non-WT) to fluconazole and voriconazole, respectively. Sixteen sequence types (STs) were detected by comparing the amino acid sequence polymorphisms of the *ERG11* gene. Fifteen isolates of STs 9, 10, 12, 13, 14, 15 and 16, were all assigned to be non-WT to fluconazole and voriconazole. By microsatellite analysis, 40 different genotypes were identified. Thirty-seven isolates from one hospital (Z1) shared the same *ERG11* sequence type (ST 2), microsatellite genotype (PU40) and drug resistance pattern. In conclusion, this is the first molecular epidemiology study of *C. guilliermondii* in China. The rate of non-WT isolates to azoles was high and the accurate contribution of *ERG11* gene mutations to azole resistance need be confirmed by further studies.

## Introduction

Invasive candidiasis is a major threat to the health of patients in hospitals, and is widely recognized as a major cause of infection-related morbidity and mortality^1, 2^. Although *Candida albicans* remains the predominant agent responsible for fungal infections, non-*albicans Candida* species are increasingly encountered^3–5^. Among these fungi, the incidence of candidaemia due to *Candida guilliermondii* ranges from 1% to 3%, depending on the geographic region^6, 7^. However, despite the low incidence of candidaemia caused by this organism, *C. guilliermondii* is of particular clinical significance as it exhibits increased resistance to antifungal agents, compared to other *Candida* species^6^.

*C. guilliermondii* is usually regarded as an opportunistic pathogen that is widely distributed in the natural environment, and the human skin and mucosal microflora^8^. However, this organism has been reported to be an important pathogen causing a variety of deep-seated infections in immunocompromised patients^8–10^. As such, accurate identification of this organism and determination of antifungal susceptibility profiles, is important in clinical decision making. In a previous study, we demonstrated that matrix-assisted laser desorption ionization-time of flight mass spectrometry (MALDI-TOF MS)-based systems performed much better than conventional phenotypic method (Vitek 2 Compact) for the routine identification of clinical *C. guilliermondii* isolates. In addition, reduced azole susceptibility and cross-resistance to azoles among *C. guilliermondii* isolates has been reported in our national surveillance system^11^. Thus monitoring the epidemiological changes and studying the drug resistance mechanism of this organism is important for clinical therapy decision making and infection control strategies.

Fluconazole prevents fungal cell growth by inhibiting 14-α-demethylase, an enzyme required for the production of an ergosterol precursor, and is encoded by the gene *ERG11* in *Candida* spp. Several mutations of the *ERG11* gene have been associated with fluconazole resistance in *Candida albicans, Candida parapsilosis, Candida krusei* and *Candida tropicalis* ^12–15^. However, little is known about the mechanism of fluconazole resistance in *C. guilliermondii*. Thus in the current study, we investigated one azole resistance mechanism by sequencing the *ERG11* gene of 164 *C. guilliermondii* isolates collected from a nationwide multi-center surveillance program called China Hospital Invasive Fungal Surveillance Net (CHIF-NET)^4^. Additionally, we performed microsatellite analysis to determine whether isolates with shared mutations originated from a shared lineage.

## Results

### Geographic distribution for *C. guilliermondii* isolates

**Figure 1:**
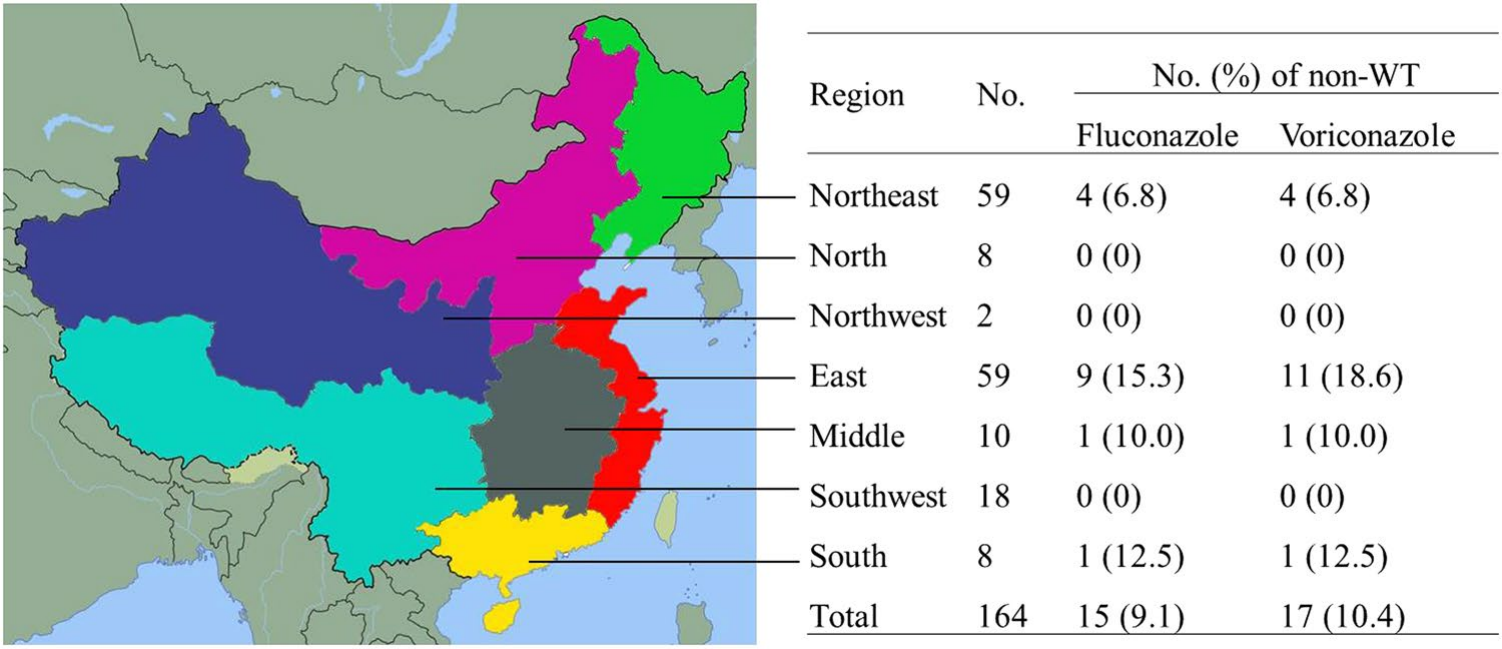
Geographical distribution of 164 *C. guilliermondii* and non-WT to fluconazole and voriconazole isolates. Different colors represent different regions of China. Green, Northeast; Orange, North; Blue, Northwest; Red, East; Grey, Middle; SkyBlue, Southwest; Yellow, South. The map was generated by GNU Image Manipulation Program (version 2.8.14, the GIMP Team, USA). The copyright holder grants anyone the right to use this work for any purpose, without any conditions, unless such conditions are required by law. Please refer the website below to see detailed information. https://commons.wikimedia.org/wiki/File:China_blank_map.svg.

Most of the studied isolates originated from the northeastern (36%, 59 isolates) and eastern (36%, 59 isolates) parts of China. About 11% of the isolates (18 of 164) were collected from southwest China, and only a small number from each of the other regions (Fig. 1). Of the 59 isolates from northeast of China, the majority (62.7%; 37/59) originated from one hospital (Z1; The first hospital of China medical university), which is a large teaching university hospital with more than two thousand hospital beds. The remaining isolates were distributed sporadically amongst 36 hospitals (1 to 11 isolates per hospital).

### Antifungal susceptibility of *C. guilliermondii* isolates

For the 164 *C. guilliermondii* isolates studied, the mean MICs for fluconazole and voriconazole were 4.18 μg/ml and 0.14 μg/mL, the MIC_50_ for fluconazole and voriconazole were 4 μg/mL and 0.12 μg/mL, and the MIC_90_ for fluconazole and voriconazole were 8 μg/mL and 0.25 μg/mL, respectively. Fifteen (9.1%) and 17 (10.4%) isolates were assigned to be non-wild type (non-WT) to fluconazole and voriconazole, respectively. Only 2 isolates were assigned to be non-WT to voriconazole but wild type (WT) to fluconazole. The non-WT strains to fluconazole were isolated from south (12.5%, 1/8), east (15.3%, 9/59), middle (10.0%, 1/10), and northeast (6.8%, 4/59) parts of the country (Fig. 1).

### Sequencing of *ERG11*

The *ERG11* gene was amplified and sequenced in each of the 164 isolates. The *C. guilliermondii* isolates were classified into 16 sequence types (STs) which were designated ST 1-ST 16 as per the amino acid sequence polymorphisms of the *ERG11* gene (Supplementary Fig. 1). The amino acid mutations were identified by comparing with the most frequent genotype, ST 1. The relationship among STs, drug resistance rates, and *ERG11* mutations are shown in Table 1. STs 1, 2, 3, 4 and 5 were present in 39.6% (65 isolates), 27.4% (45 isolates), 15.2% (25 isolates), 3.7% (6 isolates) and 1.8 (3 isolates) of the 164 isolates, respectively. The remaining 20 isolates were distributed sporadically and classified into the other 11 STs (Table 1).

**Table 1:** Results of *in vitro* azole susceptibility testing and 16 *ERG11* sequence type analysis for 164 clinical isolates of *Candida guilliermondii*.

Sequence type	No.	%	No. (%) of non-WT isolates		Non-synonymous mutations of *ERG11*
			Fluconazole	Voriconazole	
1	65	39.6	1 (1.5)	1 (1.5)	Reference sequence
2	45	27.4	0 (0)	0 (0)	W37C
3	25	15.2	0 (0)	0 (0)	P518R
4	6	3.7	0 (0)	0 (0)	P430Q
5	3	1.8	0 (0)	0 (0)	D492N, P518R
6	1	0.6	0 (0)	0 (0)	W37C, P518R
7	1	0.6	0 (0)	0 (0)	Y41F, L328T, S346T, V410M, S420T, N485K
8	3	1.8	1 (33.3)	3 (100)	Y41F, L328T, S346T, V410M, S420T
9	4	2.4	4 (100)	4 (100)	Y41F, Y132F, L328T, S346T, V410M, S420T
10	1	0.6	1 (100)	1 (100)	G16S, Y41F, Y132F, M332I, S346T, S420T
11	2	1.2	0 (0)	0 (0)	G16S, F39L, R247K, L328I, S346T, S420T
12	2	1.2	2 (100)	2 (100)	Y41F, L328T, S346T, V410M, S420T, G459S
13	2	1.2	2 (100)	2 (100)	Y132F
14	2	1.2	2 (100)	2 (100)	K143R, P518R
15	1	0.6	1 (100)	1 (100)	Q469K
16	1	0.6	1 (100)	1 (100)	I303V
Total	164	100	15 (9.1)	17 (10.4)	

Only one isolate of the 65 ST 1 *C. guilliermondii* isolates was assigned to be non-WT to fluconazole and voriconazole. ST 2, 3, 4, 5, 6, 7, 11 isolates (n = 83) were assigned to be WT to fluconazole and voriconazole. ST 9, 10, 12, 13, 14, 15, 16 isolates (n = 15) were all assigned to be non-WT to both azoles. Among the three ST 8 isolates, all were non-WT to voriconazole but only one isolate was non-WT to fluconazole.

Nineteen point mutations of the amino acid sequence of *ERG11* gene were identified by comparing with the sequence of ST 1. ST 2, 3, 4, 13, 15, and 16, were characterized by single point mutations W37C, P518R, P430Q, Y132F, Q469K and I303V, respectively. STs 5, 6 and 14 had double mutations, D492N, P518R; W37C, P518R; and K143R, P518R. The remaining STs had multi-site mutations. Isolates with Y132F (STs 9, 10 and 13) mutation were all non-WT to both azoles.

### Microsatellite analysis

**Figure 2:**
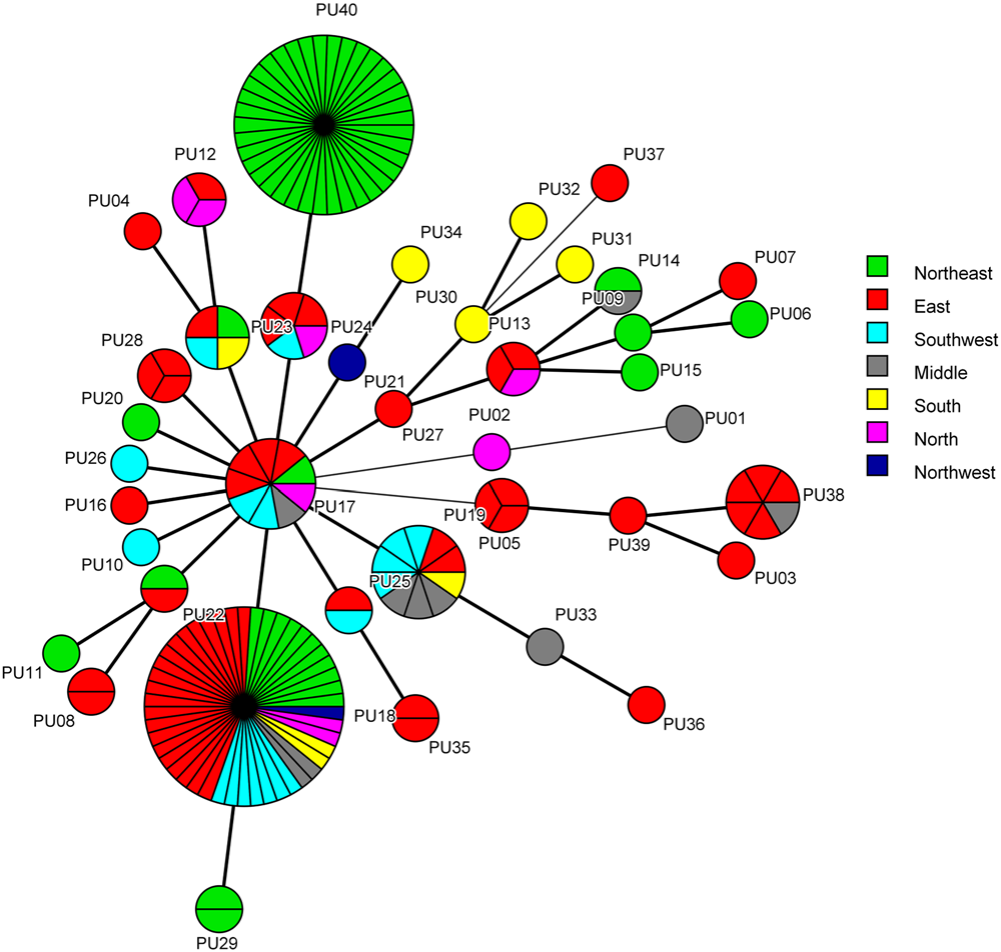
Geographical distribution of the different microsatellite genotypes in China. Minimum spanning tree analysis based on the three loci of microsatellite data. Each circle corresponds to a microsatellite genotype. Different colors represent different regions of China. Green, Northeast; Orange, North; Blue, Northwest; Red, East; Grey, Middle; SkyBlue, Southwest; Yellow, South. The lines between circles indicate the similarity between profiles: bold line, 2 of 3 microsatellite loci in common; normal line, 1 locus in common.

Using three loci for microsatellite analysis, namely sc15, sc32 and sc72, we identified 9, 6 and 21 different alleles, amongst 164 isolates, respectively. By combination analysis of the three loci, 40 different genotypes were identified, designated PU01-PU40, of which 23 were observed only once (Fig. 2). The most prevalent genotype was PU18 (n = 46, 28%), followed by PU40 (n = 37, 22.6%), PU19 (n = 10, 6.1%), and PU17 (n = 9, 5.5%). The 37 isolates from hospital Z1 (Northeast of China) shared the same genotype (PU40), which may be a clonal transmission or outbreak within the hospital (Fig. 2). The other genotypes were dispersedly distributed among different hospitals and regions.

**Figure 3:**
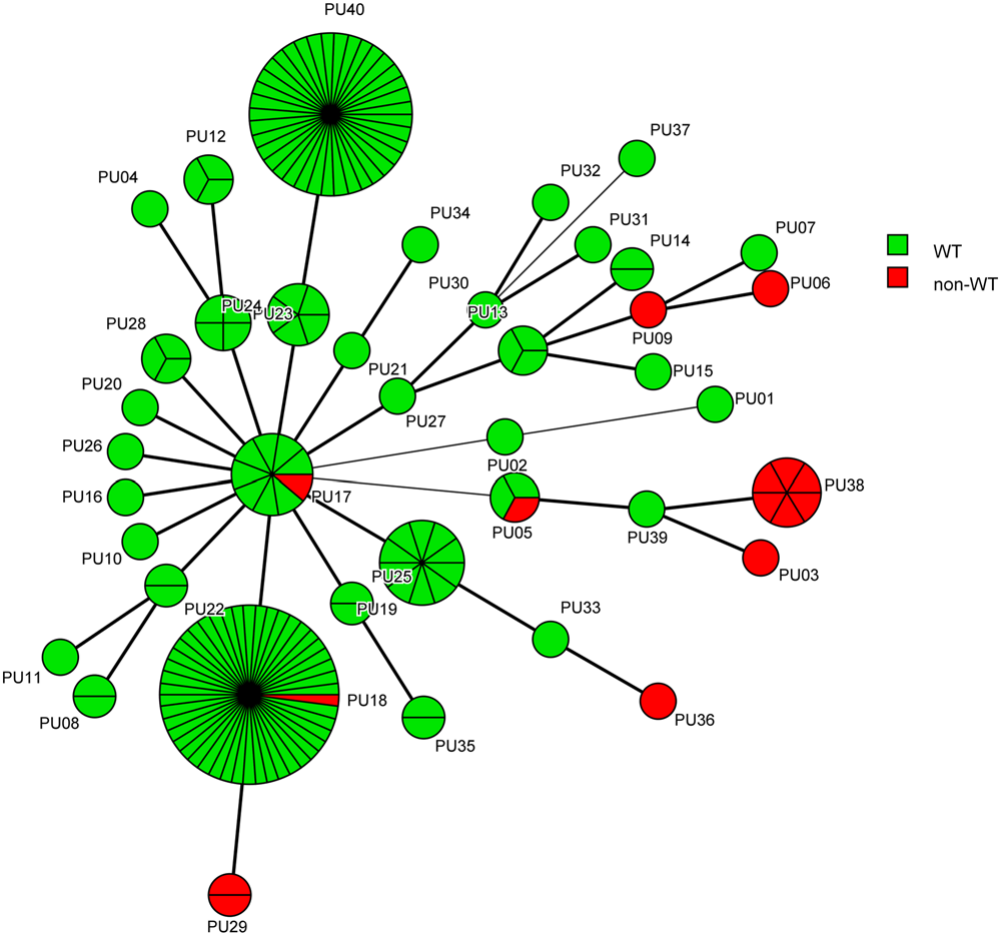
The association between microsatellite genotypes and antifungal susceptibility patterns of fluconazole of *C. guilliermondii*. Minimum spanning tree analysis based on the three loci of microsatellite data. Each circle corresponds to a microsatellite genotype. Different circle colors represent drug resistance pattern of fluconazole; Green, WT; Red, non-WT. The lines between circles indicate the similarity between profiles: bold line, 2 of 3 microsatellite loci in common; normal line, 1 locus in common.

**Figure 4:**
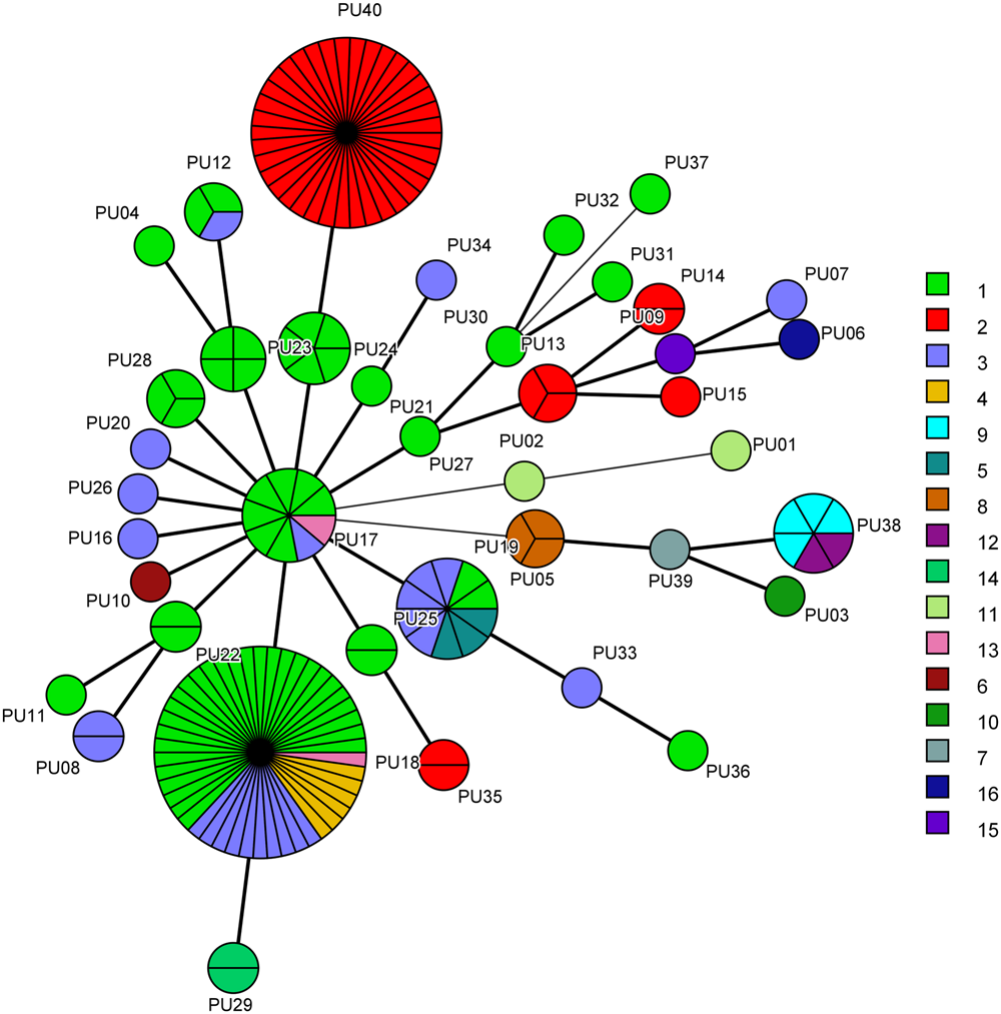
The association between microsatellite genotypes and *ERG11* STs of *C. guilliermondii*. Minimum spanning tree analysis based on the three loci of microsatellite data. Each circle corresponds to a microsatellite genotype. Different circle colors represent *ERG11* sequence types. The lines between circles indicate the similarity between profiles: bold line, 2 of 3 microsatellite loci in common; normal line, 1 locus in common.

The minimum spanning tree generated by microsatellite analysis also showed the relationship between the microsatellite genotype and drug resistance pattern. As can be seen clearly in Fig. 3, isolates of genotypes PU03, PU06, PU09, PU29, PU36 and PU38, were all non-WT to fluconazole. Furthermore, one of the 3 PU05 isolates, one of the 9 PU17 isolates, and one of the 46 PU18 isolates, were all non-WT to fluconazole. The relationship between microsatellite genotype and *ERG11* sequence type are shown in Fig. 4. STs 1, 2, and 3 were divided by different microsatellite genotypes, but some genotypes were associated with some STs, such as PU03/ST 10, PU05/ST 8, PU09/ST 15, PU10/ST 6, and PU39/ST 7.

## Discussion

*Candida guilliermondii* is an uncommon organism throughout most of the world^8^. In our study, *C. guilliermondii* isolates represented 1.7% (164/9673) of all the yeasts isolated during a five year surveillance study (CHIF-NET 2010–2014). In the present study, the majority (64.5%; 106 of 164) of the *C. guilliermondii* isolates was acquired from blood cultures, which is similar to other studies^6^, and may lead to unfavourable outcomes especially for compromised cancer hosts. The geographical distribution of the isolates varied widely, with the majority of the isolates derived from the east (36%, 59 isolates) and northeast (36%, 59 isolates) of China (Fig. 1).

Triazole antifungals are used as front-line drugs for the treatment and prophylaxis of many *Candida* infections. However, with long-term treatment, azole-resistant and cross-resistance phenotypes of *C. guilliermondii* isolates have appeared. In the global ARTEMIS DISK Antifungal Surveillance Program study (from 1997 to 2003), the resistance rates to fluconazole and voriconazole were 10.8% and 4.9%, respectively^6^. A study performed in Taiwan indicated that the non-wild type (WT) rates of both azoles is around 4%^16^. In the present study, 9.1% and 10.4% of the *C. guilliermondii* isolates were non-WT to fluconazole and voriconazole, respectively. Furthermore, some isolates showed high level minimum inhibitory concentrations (MICs) to both azoles, which is an important consideration for antifungal therapy.

Studies have been carried out to elucidate the mechanism of azole resistance in the common *Candida* species like *C. albicans, C. parapsilosis, C. krusei* and *C. tropicalis* ^12–15^. One of the major mechanisms described is the mutation of *ERG11*, the gene encoding the target of azoles in the ergosterol biosynthesis pathway, which may reduce the target affinity to fluconazole^17^. To the best of our knowledge, to date, no study has been carried out to elucidate azole resistance mechanisms in *C. guilliermondii*. By comparing the polymorphism of the *ERG11* gene, we identified 16 sequence types (STs), some of which were closely associated with antifungal susceptibility test results. Thus the possible contribution of the amino acid substitutions to azole resistance need further studies to confirm the present findings.

Microsatellite typing of *C. guilliermondii* was first established by Wrent *et al*. in 2015^18^, by combining three microsatellite markers, which delivered high discrimination, accuracy and reproducibility. In the present study, 40 genotypes were identified by microsatellite analysis, and were distributed sporadically among different regions of China. Interestingly, the 37 isolates from hospital Z1 shared the same *ERG11* sequence type (ST), microsatellite genotype and drug resistance pattern, suggesting a common origin source. To confirm whether this was an outbreak needs further studies, combining clinical information, and other data obtained from collecting environment samples, and performing further genomic analysis. A previous study reported a large pseudo-outbreak of *C. guilliermondii* fungemia at a university hospital in Brazil due to poor techniques in drawing blood samples for culture^19^. In the present study, some microsatellite genotypes were associated with drug resistance pattern, and may be an effective typing tool to explore the clonal transmission and outbreak of *C. guilliermondii*.

There are several limitations to this study. First, we only studied one possible azole resistance mechanism; studying other possible mechanisms, including genes associated with up-regulation of drug efflux pumps, and up-regulation of *ERG11* or other potential mechanisms, would have yielded more information which would have helped in coming up with firmer conclusions. Second, we did not perform further experiments to confirm that the mutations we described could confer resistance to a susceptible isolate.

In conclusion, this is the first molecular epidemiology study of *C. guilliermondii* in China. The rates of non-WT isolates to azoles were high and the contribution of *ERG11* gene mutations to azole resistance need to be confirmed by further studies.

## Methods

### Ethics statement

All methods were carried out in accordance with the guidelines of PUMCH. The study was approved by the Human Research Ethics Committee of PUMCH (S-263). Written informed consent was obtained from patients for the use of the samples in research.

### Yeast isolates

A total of 164 non-duplicate *C. guilliermondii* isolates collected from 37 hospitals distributed in 18 provinces across China during the period 2010–2014, were included in the study, which were stored at ultra-low temperature freezer before use. All the isolates were obtained from blood, ascitic fluid, peritoneal fluid, catheter, pus or other sterile body fluids. Strains were reactivated by inoculating onto Sabouraud dextrose agar for 48 h at 35 °C.

### DNA extraction and identification

DNA extraction and amplification of the ITS region was performed with primer pairs ITS1/ITS4, as previously described^20^. The PCR products were sequenced in both directions using the DNA analyzer ABI 3730XL system (Applied Biosystems, Foster City, CA). Identification was carried out by querying the sequences against GenBank database with nucleotide Basic Local Alignment Search Tool (BLASTn, http://blast.ncbi.nlm.nih.gov).

### Antifungal susceptibility testing

The *in vitro* susceptibility to fluconazole and voriconazole was determined by the broth microdilution method according to Clinical and Laboratory Standards Institute (CLSI) guidelines (document M27-A4)^21^. Minimum inhibitory concentration (MIC) values for *C. guilliermondii* isolates were interpreted according to the epidemiological cut-off values (ECVs) previously published by Pfaller *et al*.^22^ as follows: wild-type (WT), MIC of ≤8 μg/ml (fluconazole) and ≤0.25 μg/ml (voriconazole); non-WT, MIC >8 μg/ml (fluconazole) and >0.25 μg/ml (voriconazole). *C. parapsilosis* ATCC 22019 and *C. krusei* ATCC 6258 were used as quality control strains.

### Sequencing of *ERG11*

The *ERG11* gene was amplified by PCR using the following primers: ERGF with ERGR, and sequencing was performed by three primers, ERGF, ERGR and ERGA (Table 2), which were designed by Primer Premier 5.0 software (Premier, Canada). PCR conditions were as follows: 95 °C for 5 min; 95 °C for 40 s, 50 °C for 40 s, and 72 °C for 90 s, 35 times; followed by an extension step at 72 °C for 5 min. The PCR products were sequenced using an ABI 3730 sequencer (Applied Biosystems, Foster City, CA). After this, the sequences of the 1569 bp length *ERG11* gene for each isolate were determined.

**Table 2:** Primers used for amplification and sequencing.

Gene	Sequence (5′-3′)
ERG11-F	TAAGCGACCGTATGTGAG
ERG11-R	CGAGGCTGGTACTTTGAT
ERG11-A	ATGGAACAGAAAAAGTTTGCCA
sc15-F	AGGAAATGGTGGACGACAAG
sc15-R	TGAGTAGGGCTTGCACACTG
sc32-F	GCGTCCTTATCGTCTTCGTC
sc32-R	ATGGGTGGATATCGTGGAAA
sc72-F	ACCATAGAATGAGCGGTAGCA
sc72-R	TTTCTGTTCCAAGGCCAAAG
M13	TGTAAAACGACGGCCAGT

### Microsatellite amplification and analysis

A panel of three short tandem repeat (STR) markers (sc15, sc32 and sc72) was used for genotyping the *C. guilliermondii* isolates as previously described by Wrent *et al*.^18^. Each specific forward primer was 5′-tailed with the M13 universal sequence and the universal M13 primer was labeled with the fluorescent dye FAM-6. The program included 1 cycle of 1 min at 94 °C, 10 cycles of 30 s at 94 °C, 30 s at 60 °C (after each cycle the annealing temperature was decreased by 1 °C), and 30 s at 72 °C; then 20 cycles of 30 s at 94 °C, 30 s at 50 °C, and 30 s at 72 °C; and a final extension step of 2 min at 72 °C. The microsatellite PCR product was measured on an ABI 3730 DNA analyzer (Applied Biosystems, Foster City, CA,USA) using the GeneScan™ 500 LIZ® Size Standard marker 30–600 bp (Life Technologies). The results were analyzed by GeneMarker software (Version 2.2.0, Soft Genetics, State College, PA, USA). Repeat numbers of the three loci were analyzed using BioNumerics software v6.5 (Applied Maths, Texas, USA) for cluster analysis. A minimum spanning tree was constructed using the unweighted-pair group method with arithmetic mean clustering (UPGMA), treating the data as categorical information.

## Electronic supplementary material

Supplementary Information

## References

[1] Pfaller MA, Diekema DJ (2007). Epidemiology of invasive candidiasis: a persistent public health problem. Clin Microbiol Rev.

[2] Kullberg BJ, Arendrup MC (2015). Invasive candidiasis. N Engl J Med.

[3] Miceli MH, Diaz JA, Lee SA (2011). Emerging opportunistic yeast infections. Lancet Infect Dis.

[4] Wang H (2012). *In vitro* susceptibilities of yeast species to fluconazole and voriconazole as determined by the 2010 national China Hospital Invasive Fungal Surveillance Net (CHIF-NET) study. J Clin Microbiol.

[5] Pfaller MA (2010). Results from the ARTEMIS DISK Global Antifungal Surveillance Study, 1997 to 2007: a 10.5-year analysis of susceptibilities of *Candida* Species to fluconazole and voriconazole as determined by CLSI standardized disk diffusion. J Clin Microbiol.

[6] Pfaller MA (2006). *Candida guilliermondii*, an opportunistic fungal pathogen with decreased susceptibility to fluconazole: geographic and temporal trends from the ARTEMIS DISK antifungal surveillance program. J Clin Microbiol.

[7] Pfaller M (2012). Epidemiology and outcomes of candidemia in 3648 patients: data from the Prospective Antifungal Therapy (PATH Alliance(R)) registry, 2004–2008. Diagn Microbiol Infect Dis.

[8] Savini V (2011). What do we know about *Candida guilliermondii*? A voyage throughout past and current literature about this emerging yeast. Mycoses.

[9] Papon N (2013). *Candida guilliermondii*: biotechnological applications, perspectives for biological control, emerging clinical importance and recent advances in genetics. Curr Genet.

[10] Savini V (2010). Pan-azole-Resistant *Candida guilliermondii* from a Leukemia Patient’s Silent Funguria. Mycopathologia.

[11] Cheng, J. W. *et al*. Identification and antifungal susceptibility profile of *Candida guilliermondii* and *Candida fermentati* from a multi-center study in China. *J Clin Microbiol ***54**, 2187–2189. doi:10.1128/JCM.00938-16 (2016). 10.1128/JCM.00938-16 PMC4963529 27252461

[12] Forastiero A (2013). *Candida tropicalis* antifungal cross-resistance is related to different azole target (Erg11p) modifications. Antimicrob Agents Chemother.

[13] Berkow EL (2015). Multidrug Transporters and Alterations in Sterol Biosynthesis Contribute to Azole Antifungal Resistance in *Candida parapsilosis*. Antimicrob Agents Chemother.

[14] Flowers SA, Colon B, Whaley SG, Schuler MA, Rogers PD (2015). Contribution of clinically derived mutations in *ERG11* to azole resistance in *Candida albicans*. Antimicrob Agents Chemother.

[15] Ricardo E (2014). *In vivo* and *in vitro* acquisition of resistance to voriconazole by *Candida krusei*. Antimicrob Agents Chemother.

[16] Chen CY (2013). Clinical features of patients with infections caused by *Candida guilliermondii* and *Candida fermentati* and antifungal susceptibility of the isolates at a medical centre in Taiwan, 2001–10. J Antimicrob Chemother.

[17] Sanglard D, Odds FC (2002). Resistance of *Candida* species to antifungal agents: molecular mechanisms and clinical consequences. Lancet Infect Dis.

[18] Wrent P, Rivas EM, Peinado JM, de Siloniz MI (2016). Development of an affordable typing method for *Meyerozyma guilliermondii* using microsatellite markers. Int J Food Microbiol.

[19] Medeiros EA (2007). Evidence for a pseudo-outbreak of *Candida guilliermondii* fungemia in a university hospital in Brazil. J Clin Microbiol.

[20] Zhang L (2014). Yeast identification algorithm based on use of the Vitek MS system selectively supplemented with ribosomal DNA sequencing: proposal of a reference assay for invasive fungal surveillance programs in China. J Clin Microbiol.

[21] CLSI. Reference Method for Broth Dilution Antifungal Susceptibility Testing of Yeasts; Approved Standard -Third Edition. CLSI document M27-A4 (Wayne, PA: Clinical and Laboratory Standards Institute; 2008).

[22] Pfaller MA, Diekema DJ (2012). Progress in antifungal susceptibility testing of *Candida* spp. by use of Clinical and Laboratory Standards Institute broth microdilution methods, 2010 to 2012. J Clin Microbiol.

