# Supplementary material for: Molecular epidemiology and azole resistance mechanism study of Candida guilliermondii from a Chinese surveillance system
Source: Sci Rep. 2017 Apr 19;7:907. doi: 10.1038/s41598-017-01106-7 (PMC5430413; doi:10.1038/s41598-017-01106-7)
Supplement: Supplementary file 1 — Supplementary Information [file 41598_2017_1106_MOESM1_ESM.pdf]

Molecular epidemiology and azole resistance mechanism study of *Candida guilliermondii* from a Chinese surveillance system

Jing-Wei Cheng[1, 2, 3]†, Kang Liao[4,]†, Timothy Kudinha[5, 6], Shu-Ying Yu[1, 2, 3], Meng Xiao[1, 2, 3], He Wang[1, 2, 3], Fanrong Kong[5], Ying-Chun Xu[1, 2, 3,*]

[1] Department of Clinical Laboratory, Peking Union Medical College Hospital, Chinese Academy of Medical Sciences, Beijing 100730, China

[2] Graduate School, Peking Union Medical College, Chinese Academy of Medical Sciences, Beijing 100730, China

[3] Beijing Key Laboratory for Mechanisms Research and Precision Diagnosis of Invasive Fungal Diseases, Beijing 100730, China

[4] Department of Laboratory Medicine, The First Affiliated Hospital of Sun Yat-sen University, Guangzhou, Guangdong 510080, China

[5] The Charles Sturt University, Leeds Parade, Orange, New South Wales 2687, Australia

[6] Centre for Infectious Diseases and Microbiology Laboratory Services, Westmead Hospital, Westmead, New South Wales 2145, Australia

†These authors contributed equally to this work.

***Corresponding author:** Prof. Ying-Chun Xu, xycpumch@139.com

Supplementary Fig. 1 The 16 sequence types (STs) designated ST 1-ST 16 as per the amino acid sequence polymorphisms of the *ERG11* gene

```
                     20                    40                    60                    80
1  MAIADIATWV YSEFRGLPVS AQISIVVLFP FVYNLLWQFW YSLQKDRAPL VFHWVPWLGS SVVYSMQPYE FFESCRQKYG DVFSFVMVGR
2  .......... .......... .......... ......C... .......... .......... .......... .......... ..........
3  .......... .......... .......... .......... .......... .......... .......... .......... ..........
4  .......... .......... .......... .......... .......... .......... .......... .......... ..........
5  .......... .......... .......... .......... .......... .......... .......... .......... ..........
6  .......... .......... .......... ......C... .......... .......... .......... .......... ..........
7  .......... .......... .......... .......... F......... .......... .......... .......... ..........
8  .......... .......... .......... .......... F......... .......... .......... .......... ..........
9  .......... .......... .......... .......... F......... .......... .......... .......... ..........
10 .......... .....S.... .......... .......... F......... .......... .......... .......... ..........
11 .......... .....S.... .......... ........L. .......... .......... .......... .......... ..........
12 .......... .......... .......... .......... F......... .......... .......... .......... ..........
13 .......... .......... .......... .......... .......... .......... .......... .......... ..........
14 .......... .......... .......... .......... .......... .......... .......... .......... ..........
15 .......... .......... .......... .......... .......... .......... .......... .......... ..........
16 .......... .......... .......... .......... .......... .......... .......... .......... ..........

          100                   120                   140                   160                   180
1  VMTVYLGPKG HEFVFNSKAT DVSAEDAYTH LTTPVFGKGV IYDCPNSRLM EQKKFAKFAL TKDSFIKYVP KIQDEVLSYF VEHDFKMKEK
2  .......... .......... .......... .......... .......... .......... .......... .......... ..........
3  .......... .......... .......... .......... .......... .......... .......... .......... ..........
4  .......... .......... .......... .......... .......... .......... .......... .......... ..........
5  .......... .......... .......... .......... .......... .......... .......... .......... ..........
6  .......... .......... .......... .......... .......... .......... .......... .......... ..........
7  .......... .......... .......... .......... .......... .......... .......... .......... ..........
8  .......... .......... .......... .......... .......... .......... .......... .......... ..........
9  .......... .......... .......... .......... .F........ .......... .......... .......... ..........
10 .......... .......... .......... .......... .F........ .......... .......... .......... ..........
11 .......... .......... .......... .......... .......... .......... .......... .......... ..........
12 .......... .......... .......... .......... .......... .......... .......... .......... ..........
13 .......... .......... .......... .......... .F........ .......... .......... .......... ..........
14 .......... .......... .......... .......... .......... ..R....... .......... .......... ..........
15 .......... .......... .......... .......... .......... .......... .......... .......... ..........
16 .......... .......... .......... .......... .......... .......... .......... .......... ..........

                    200                   220                   240                   260
1  DSGVVNVMKT QPEITIFTAS RSLMGDEMRK RFDTSFAQLY SDLDKGFHPI HFAFPHLPLP MSRNRDRAQQ KISATYKGLI DTRRSSGNID
2  .......... .......... .......... .......... .......... .......... .......... .......... ..........
3  .......... .......... .......... .......... .......... .......... .......... .......... ..........
4  .......... .......... .......... .......... .......... .......... .......... .......... ..........
5  .......... .......... .......... .......... .......... .......... .......... .......... ..........
6  .......... .......... .......... .......... .......... .......... .......... .......... ..........
7  .......... .......... .......... .......... .......... .......... .......... .......... ..........
8  .......... .......... .......... .......... .......... .......... .......... .......... ..........
9  .......... .......... .......... .......... .......... .......... .......... .......... ..........
10 .......... .......... .......... .......... .......... .......... .......... .......... ..........
11 .......... .......... .......... .......... .......... .......... ......K... .......... ..........
12 .......... .......... .......... .......... .......... .......... .......... .......... ..........
13 .......... .......... .......... .......... .......... .......... .......... .......... ..........
14 .......... .......... .......... .......... .......... .......... .......... .......... ..........
15 .......... .......... .......... .......... .......... .......... .......... .......... ..........
16 .......... .......... .......... .......... .......... .......... .......... .......... ..........

          280                   300                   320                   340                   360
1  PTRDLIDSLM ATSTYKDGVK MTDQEIANLL IGILMGGQHT SAATSAWFLL HLGERPDLQE EMYKEICGVF EKKNKSFKDL SYDDLQEMPL
2  .......... .......... .......... .......... .......... .......... .......... .......... ..........
3  .......... .......... .......... .......... .......... .......... .......... .......... ..........
4  .......... .......... .......... .......... .......... .......... .......... .......... ..........
5  .......... .......... .......... .......... .......... .......... .......... .......... ..........
6  .......... .......... .......... .......... .......... .......... .......... .......... ..........
7  .......... .......... .......... .......... .......... .......T.. .......... .....T.... ..........
8  .......... .......... .......... .......... .......... .......T.. .......... .....T.... ..........
9  .......... .......... .......... .......... .......... .......T.. .......... .....T.... ..........
10 .......... .......... .......... .......... .......... .......... .I........ .....T.... ..........
11 .......... .......... .......... .......... .......... .......I.. .......... .....T.... ..........
12 .......... .......... .......... .......... .......... .......T.. .......... .....T.... ..........
13 .......... .......... .......... .......... .......... .......... .......... .......... ..........
14 .......... .......... .......... .......... .......... .......... .......... .......... ..........
15 .......... .......... .......... .......... .......... .......... .......... .......... ..........
16 .......... .......... .......... ..V....... .......... .......... .......... .......... ..........

                    380                   400                   420                   440
1  VNNVIKETLR MHMPLHSIFR KVKKPLLVPG TKYTVPKGHY VMVSPGYAMV NDRWFPDAAS FNPRRWDEQP LPTDGQDETV DYGFGNISKG
2  .......... .......... .......... .......... .......... .......... .......... .......... ..........
3  .......... .......... .......... .......... .......... .......... .......... .......... ..........
4  .......... .......... .......... .......... .......... .......... .........Q .......... ..........
5  .......... .......... .......... .......... .......... .......... .......... .......... ..........
6  .......... .......... .......... .......... .......... .......... .......... .......... ..........
7  .......... .......... .......... .......... .........M .........T .......... .......... ..........
8  .......... .......... .......... .......... .........M .........T .......... .......... ..........
9  .......... .......... .......... .......... .........M .........T .......... .......... ..........
10 .......... .......... .......... .......... .......... .........T .......... .......... ..........
11 .......... .......... .......... .......... .......... .........T .......... .......... ..........
12 .......... .......... .......... .......... .........M .........T .......... .......... ..........
13 .......... .......... .......... .......... .......... .......... .......... .......... ..........
14 .......... .......... .......... .......... .......... .......... .......... .......... ..........
15 .......... .......... .......... .......... .......... .......... .......... .......... ..........
16 .......... .......... .......... .......... .......... .......... .......... .......... ..........
```

```
                   460                 480                 500                 520
                     |                   |                   |                   |
 1 VSSPYLPFGG GRHRCIGEQF AYVQLGTILA SYVYNVTWEL KDKLPGVDYA SMVTLPLEPA DIVWKKRPTC VFX 523
 2 .......... .......... .......... .......... .......... .......... .......... ... 523
 3 .......... .......... .......... .......... .......... .......... .......R.. ... 523
 4 .......... .......... .......... .......... .......... .......... .......... ... 523
 5 .......... .......... .......... .......... .N........ .......... .......R.. ... 523
 6 .......... .......... .......... .......... .......... .......... .......R.. ... 523
 7 .......... .......... .......... ....K..... .......... .......... .......... ... 523
 8 .......... .......... .......... .......... .......... .......... .......... ... 523
 9 .......... .......... .......... .......... .......... .......... .......... ... 523
10 .......... .......... .......... .......... .......... .......... .......... ... 523
11 .......... .......... .......... .......... .......... .......... .......... ... 523
12 ........S. .......... .......... .......... .......... .......... .......... ... 523
13 .......... .......... .......... .......... .......... .......... .......... ... 523
14 .......... .......... .......... .......... .......... .......... .......R.. ... 523
15 .......... ........K. .......... .......... .......... .......... .......... ... 523
16 .......... .......... .......... .......... .......... .......... .......... ... 523
```
